# Preparations for a European R&D roadmap for an inertial fusion demo reactor

**DOI:** 10.1098/rsta.2020.0005

**Published:** 2020-12-07

**Authors:** P. A. Norreys, L. Ceurvorst, J. D. Sadler, B. T. Spiers, R. Aboushelbaya, M. W. Mayr, R. Paddock, N. Ratan, A. F. Savin, R. H. W. Wang, K. Glize, R. M. G. M. Trines, R. Bingham, M. P. Hill, N. Sircombe, M. Ramsay, P. Allan, L. Hobbs, S. James, J. Skidmore, J. Fyrth, J. Luis, E. Floyd, C. Brown, B. M. Haines, R. E. Olson, S. A. Yi, A. B. Zylstra, K. Flippo, P. A. Bradley, R. R. Peterson, J. L. Kline, R. J. Leeper

**Affiliations:** 1 Department of Physics, University of Oxford, Oxford, UK; 2 UKRI-STFC Central Laser Facility, Didcot, UK; 3 CELIA, Université de Bordeaux-CNRS-CEA, Talence, France; 4 Los Alamos National Laboratory, Los Alamos, NM, USA; 5 University of Strathclyde, Glasgow, UK; 6 Atomic Weapons Establishment, Aldermaston, UK

**Keywords:** inertial fusion energy, inertial confinement fusion, high-energy density plasma physics, fast ignition, auxiliary heating, IFE Roadmap

## Abstract

A European consortium of 15 laboratories across nine nations have worked together under the EUROFusion Enabling Research grants for the past decade with three principle objectives. These are: (a) investigating obstacles to ignition on megaJoule-class laser facilities; (b) investigating novel alternative approaches to ignition, including basic studies for fast ignition (both electron and ion-driven), auxiliary heating, shock ignition, etc.; and (c) developing technologies that will be required in the future for a fusion reactor. A brief overview of these activities, presented here, along with new calculations relates the concept of auxiliary heating of inertial fusion targets, and provides possible future directions of research and development for the updated European Roadmap that is due at the end of 2020.

This article is part of a discussion meeting issue ‘Prospects for high gain inertial fusion energy (part 2)’.

## 1. Introduction

In early March 2020, a Hooke Discussion Meeting entitled ‘Prospects for high gain inertial fusion energy’ was held at The Royal Society in London. Eighty fusion scientists from around the globe met to discuss the possibility of producing net electrical power from the nuclear fusion reactions that take place inside igniting nuclear fuel at the extremes of density, temperature and electromagnetic radiation. The goal of this initiative is to prepare an internationally endorsed Roadmap for an Inertial Fusion European Demonstration Reactor and for accelerating the development of the associated safeguards and the potentially ‘transformative’ technology. The Roadmap itself builds upon Enabling Research grants from EUROFusion, part of EURATOM, from 2014–2018 (Principal Investigator: Sylvie Jacquemot) and 2019–2020 (Principal Investigator: Peter Norreys) awarded to a European consortium comprising 15 laboratories across nine nations, including the UK. The grants have supported preparations for integrated experiments on the PETAL/LMJ facility in Bordeaux, France, and have focused on advancing inertial fusion energy science via microphysics studies using existing high-power laser facilities. These include the UKRI-STFC Central Laser Facility’s Vulcan laser at the Rutherford Appleton Laboratory, the LULI2000 laser facility at Ecole Polytechnique, the PALS laser facility in the Czech Republic and other newly commissioned laser systems across Europe (e.g. the CALA laser facility in Germany, the FLAME laser facility in Frascati, Italy etc.).

Our consortium has had three principle objectives over the past decade. These are: (a) investigating obstacles to ignition on megaJoule-class laser facilities; (b) investigating novel alternative approaches to ignition, including basic studies for fast ignition (both electron and ion-driven), auxiliary heating, shock ignition, etc.; and (c) developing technologies that will be required in the future for a fusion reactor. Inertial fusion energy requires the 1000-fold compression of matter to ultra-high densities and temperatures to mimic the compressional effect of gravity in the sun, nature’s very effective nuclear fusion reactor.

By irradiating and imploding a small spherical shell containing isotopes of hydrogen fuel (deuterium and tritium) in the laboratory, the fuel’s own inertia, i.e. the tendency of matter to resist sudden acceleration, permits a sufficient fraction of the isotope pairs to fuse into a helium nucleus during the compression and stagnation stages before the fuel eventually expands from the increasingly high pressure. In ordinary circumstances, the tendency of ‘like charges to repel’ causes two positively charged hydrogen nuclei to aggressively repel each other. However, when two hydrogen nuclei are sufficiently close, even for this extremely short duration, the short-range strong nuclear force dominates over this tendency to repel, and the hydrogen nuclei can be induced to fuse into a helium nucleus. During each fusion event, a sudden and intense release of energy occurs because the rest mass of the fusion products (a helium nucleus and a free neutron) is less than the combined masses of the deuterium and tritium ions. If these fusion reactions occur at a sufficient rate, then more energy is generated than is expended to drive the initial compression of the fuel. This heat, if captured in a surrounding blanket, as in a conventional power plant, can drive a steam turbine that generates electric power.

Inertial fusion energy is the civilian spin-off arising out of the inertial confinement fusion concept associated with the nuclear weapons stockpile stewardship programmes in the USA and the UK. The concept rests upon the results of classified experiments performed under the limited UK test programme and the US Halite-Centurion programme, as well as the more recent data obtained from the National Ignition Facility at the Lawrence Livermore National Laboratory, USA. While it is true that sustained net-energy gain has not yet been achieved, sufficient scientific progress has now been made in understanding laser-driven burning plasmas to begin contemplating an energy development programme involving a next-generation facility. The US plasma physics community has also reached similar conclusions, with their community call for the establishment of an inertial fusion energy programme, published in March 2020—12 years after the commissioning of the National Ignition Facility [1].

Given the urgency of the threats facing humanity from climate change, as highlighted by Sir David Attenborough in his December 2018 speech to the UN Conference on Climate Change in Poland, such a facility should aim for net energy gain—termed ‘proof of principle’ or ‘physics break-even’, or even move towards higher gain—known as ‘engineering break-even’. We contend that if the UK community were to embark on concerted bilateral programmes with our colleagues in the USA, the EU and Asia, then we could realize this dream synergistically and expediently in a co-ordinated international effort. Given the enormous technological and scientific challenges that remain in the construction of any fusion power plant, be it inertial or magnetic, this must build upon the research now underway by the magnetic confinement fusion community in the development of integrated fusion reactor systems and associated technologies. As a result, any future European Inertial Fusion Demonstration Reactor is likely to have a similar multi-decadal timescale to realize its objectives. Therefore, the Roadmap, and the move towards a carbon-free economy, has to recognize that the mix of energy sources from the middle of the century onwards is likely to be a combination of renewable energy sources and fusion reactors (magnetic and inertial) that replace fission reactors in due course.

This paper is organized into the following sections. In the first section, the progress to date made in the indirect-drive, central hot-spot scheme at the National Ignition Facility, along with the fast ignition concept, is briefly summarized. Clearly, the driver energy requirements for sustained net energy gain are a significant factor in the capital costs for any future inertial fusion reactor and its price entry into the electricity market.

Analytic calculations indicate that the ignition threshold is close to 5 MJ if a low adiabat (*γ* ≈ 3) implosion can be achieved and 7 MJ if a higher adiabat implosion (*γ* ≈ 5/3) is realized [2]. This is corroborated by the work of Clark *et al.* [3] whose fig. 7 also indicates a capsule absorbed energy (and hence ignition threshold) is at about three times the present NIF energy.

Given that the fusion yield scales as *Y* ∼ *E*^3.3^, to achieve high gain the drive energy needs to be 20 MJ. This is consistent with a recent pulsed power driver concept that discusses the X-ray drive energy that is needed for high gain in an indirect drive hohlraum using a wire array Z-pinch device [4].

Our approach to the European conceptual demo reactor roadmap is to identify a flexible laser coupled with a pulse power driver that can be configured in a number of different irradiation geometries. The machine must be one that allows a wide range of different approaches to inertial fusion to be investigated at full energy scale. The aim is not to rule out any approach to inertial fusion before it is fully tested in the laboratory. That includes indirectly driven target designs, shock ignition, direct drive, fast ignition, impact ignition etc. This approach is similar to that of the Large Hadron Collider at the CERN laboratory, where very different high energy physics experiments use the same accelerator machine.

It is unlikely that one would want to run such a demo reactor based upon high-power laser technologies flat-out at the 20 MJ energy level, due to optical component damage, so having some leeway to safely run the facility at reduced power while retaining the capacity to access high-energy density physics conditions for ignition is helpful and, one might argue, prudent.

Consequently, the next section’s discussion focuses on alternative schemes, in particular the new concept of auxiliary heating of the central hot spot to augment the ion temperature there. The following section deals with the potential benefits of investing in an inertial fusion energy science and technology programme. The final section summarizes the paper and indicates possible scenarios that might be included in the European Roadmap that is due at the end of 2020.

## 2. Progress

### (a) Indirect drive inertial confinement fusion

The scientific and technological progress in exploratory inertial confinement fusion research in the USA has been impressive, relative to its modest government-provided research budget, over the past two decades. This is particularly true in areas related to the assembly and understanding of the high-energy-density conditions in the compressed fuel, as well as in the enabling technologies required for inertial fusion energy applications. These technologies include high-repetition-rate lasers, heavy-ion beam drivers, pulsed-power magnetic-compression generators, and high-reproducibility cryogenic-target assembly and qualification [2,5–15].

Most recently, a fusion output energy has been achieved which is more than twice the total internal kinetic energy of the imploding fuel, reaching unprecedented hot spot areal densities and stagnation pressures [14]. However, targets with high convergence ratios (the ratio of the initial radius of the fuel and ablator to its initial radius at stagnation), which are typical of central hot spot implosion capsules that have been fielded so far due to the limited energy available on the National Ignition Facility, are highly susceptible to preheat and mix. A recent study suggests that the resulting mix of ablator material and the dense fuel into the hot spot significantly raises the hot spot’s fuel adiabat (i.e. the ratio *α* of the fuel pressure to the ideal Fermi degeneracy pressure) from the designed low-level of *α* = 1.5 to an estimated α=1.9–2.9 [2,16]. This is due to the combined effects of fuel preheat, vorticity and mix of fuel and pusher (due to the Rayleigh–Taylor hydrodynamic instability and residual drive asymmetries) [17–19]. These increase the effective adiabat of the cold fuel and reduce the compressibility of the pusher, resulting in a reduction of energy delivered to the hot spot at stagnation. These values are similar to those found in higher-adiabat (the so-called ‘high-foot’) implosions, thereby limiting the potential areal density, *ρR*, and in so doing, the potential yield.

At the same time, the European consortium has worked on the underpinning microphysics including: a more precise understanding and mitigating the growth of Rayleigh–Taylor instabilities [20]; the role of non-local heat flow in burning plasmas [19] laser-plasma instabilities [21,22]; atomic physics [23,24] among others.

The experimental data are consistent with the observed trend that higher adiabat target designs perform closer to predictions than those designed for a lower adiabat. Following the first entry into this regime in 2014, there has been substantial progress in improving the fusion yield by the use of improved target and laser pulse-shape designs [14,16,18]. These advances indicate that entry into the burning plasma regime, where alpha-particle energy deposition fully dominates the hot-spot hydrodynamics, is tantalizingly close.

### (b) Fast ignition

Alternative schemes for inertial fusion attempt to circumnavigate this drive energy barrier to ignition. A notable example is the fast ignition concept that presents an opportunity to increase the efficiency of inertial fusion while simultaneously relaxing the stringency of the driver and symmetry requirements [25]. By separating the compression and ignition phases, this scheme allows for much lower implosion velocities. Thus, more fusion fuel is compressed than in the central hot spot approach for the same drive energy. This increases the achievable gain once a hot spot of ignition temperature is formed on the side of the dense fuel. Cone-guided fast ignition provides a route to minimize the distance between the compressed fuel and the required petawatt laser pulse [26,27].

A key aspect of this scheme, therefore, is the collisional deposition of energy by 1–3 MeV electron beams generated during an intense laser-plasma interaction [26–28]. In order to generate and propagate such a burn wave, analytic estimates indicate that approximately 20 k J of energy must be deposited over the course of approximately 20 p s in a spot whose radius is approximately 20 μm [29]. This stringent requirement establishes a minimum intensity (10^20^ W cm^−2^) for the ignition pulse. Assuming a reliance on collisional energy deposition, it further demands the use of frequency converted (ultra-violet) petawatt-power laser pulses, in order to keep the generated electron energy to within the collisional stopping range. This is also required in order to minimize the fast electron beam divergence, as recently indicated in the latest cone-guided fast ignition experiments on the OMEGA EP facility [29,30].

Reviews of international efforts to study the fast ignition approach are available [31,32]. The best estimates for the drive energy requirements for an ignition capsule are found in the seminal paper by Clark & Tabak [33], supported by later radiation hydrodynamic simulations by Shay *et al.* [34]. Their conclusion is that the drive energy for an isochoric fuel assembly requires 0.5–1.0 MJ drive energies for direct and indirect drive approaches, respectively. Similarly, for the energy deposition by fast electrons to raise the hot spot temperatures to ignition conditions, Strozzi *et al.* [35] found that the particle energy deposition in the hot spot is in the range of 80–130 kJ. These estimates are in reasonable agreement with Prof. Stefano Atzeni’s model [28]. When one takes into account energy losses in channel formation, cone inserts and laser-fast electron conversion efficiencies, then it is likely that a short pulse energy of similar scale to the compression drive laser is required. The drive compression energy needed to achieve the areal densities for an isochoric fuel assembly also needs to be verified in the laboratory using the National Ignition Facility and Laser MegaJoule.

The European consortium has also undertaken studies of stable channel formation in fusion relevant plasmas [36–38], electromagnetic pulse generation and mitigation [39], UV fast electron generation and energy transport [40], magnetic fields [41], reactor materials under hostile radiation environments [23], among others, including the role of magnetic fields in guiding fast electrons.

Ion-driven fast ignition is a variation on this concept and is also under investigation by our consortium. It is attracting private capital in Germany via the new company Marvel Fusion Gmbh.

Our consortium is also actively pursuing the physics of laser-plasma amplifiers as a route to overcome the need for expensive grating compressors [42,43]. There are new advances in laser technology based upon optical parametric chirped pulse amplification (OPCPA) that might also lead to multi-kJ petawatt-scale ultra-violet pulses. Despite these challenges, fast ignition promises much higher overall fusion energy gain, meaning it will likely lead to a commercially viable reactor much more quickly. Research investments in high energy ultraviolet short pulse lasers is an enabling technology that could more quickly lead to a viable commercial reactor for inertial fusion energy. It would be a fraction of the cost of one based upon the indirect drive inertial confinement fusion approach, and so is an attractive path forward for inclusion in the European Roadmap.

## 3. Auxiliary heating

We discuss here a potential solution to the two major roadblocks on the path to the realization of inertial fusion: insufficient energy in the central hot spot, and the susceptibility of high-convergence ratio implosions to hydrodynamic instabilities and drive asymmetries. Previous work has established that it is possible, in principle, for energy to be deposited into a high-density plasma via collisionless interactions at the intersection of two crossing relativistic electron beams with the maximum efficiency of deposition occurring for the case of orthogonal beams [44,45]. Crucially, unlike the collisional heating mechanisms used in fast ignition schemes, the collisionless nature of the interaction means that this auxiliary heating scheme does not disrupt the isobaric nature of a central hot-spot implosion and allows for targeted energy deposition in the hot spot rather than the surrounding dense fuel. We emphasize that auxiliary heating is at an early stage but is a promising idea.

To maximize the amount of energy deposited by the relativistic electron beams, this auxiliary heating mechanism is applied to low convergence ratio (and consequently large hot spot) implosions for the first time. For the purposes of this paper, we consider the implosions of ‘wetted foam’ capsules, a recent development in low convergence ratio target designs [46–49]. These implosions have been shown to exhibit a remarkable agreement between experimental data [47] and near one-dimensional radiation-hydrodynamics simulations using the xRAGE code, developed by Los Alamos National Laboratory [50]. While pure DT equation-of-state models tend to over-estimate the fusion yield of these targets, the inclusion of liquid DT wetted foam EOS allows the xRAGE simulations to reproduce the observed experimental variables with good accuracy, and thus enable modelling of additional energy injection into the hot spot’s thermal electron population under somewhat realistic conditions. As such, these targets provide the ideal candidate for new numerical work, presented here, demonstrating that auxiliary heating leads to an amplification in the yield of these implosions. It should be noted, however, that while the ‘wetted foam’ targets discussed here are ideal for numerical investigations the proposed scheme is flexible enough to be applied to many other capsule designs as well.

**Figure 1.**
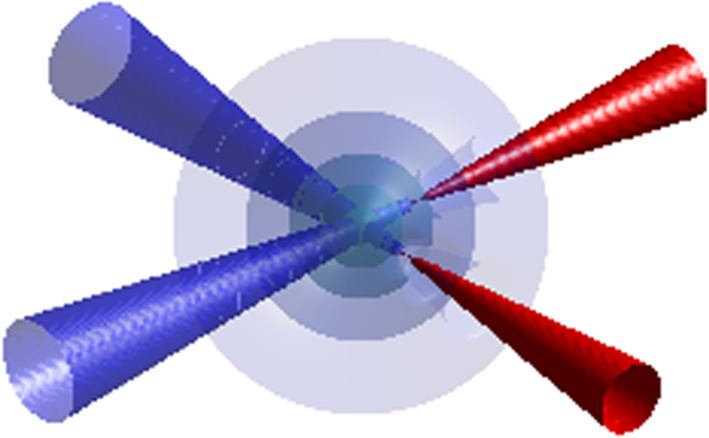
A schematic of the proposed auxiliary heating scheme, pictorially indicating the orthogonal crossing of relativistic electron beams (blue/left beams) driven by intense short-duration lasers pulses (red/right beams) in a dense plasma.(Online version in colour.)

It has been shown previously that crossing two electron beams orthogonally within a high-density, high-temperature plasma, such as is found in the hot spot, can drive Langmuir waves (longitudinal oscillations in the plasma electron density) that are unstable. This process transfers energy from the fast electron beam to the plasma electrons. Collisions that occur within the confinement time equilibrate the increased electron energy with the ion population, thereby increasing the ion temperature. A schematic of this interaction is shown in figure 1. For electron number densities on the order of 10^26^ cm^−3^ and temperatures of the order of 4 keV, Vlasov–Maxwell simulations have shown that the electron beams drive Langmuir waves to increase the plasma electrons’ kinetic energy by a factor of 2.8 and that this coupling of the relativistic electron beams to the background plasma has an efficiency of 18%. This efficiency means there is still a need for a similar short pulse energy to fast ignition, but the advantage versus fast-ignition is it can take advantage of the much more well-developed central hot-spot ignition schemes that have been extensively tested at the National Ignition Facility and Omega laser facilities using both indirect and direct drive.

In this special issue, Spiers *et al.* [38] present results of an experimental campaign on the ORION laser of the Atomic Weapons Establishment [51] that confirm the feasibility of propagating electron beams into the central hot spot of a fusion implosion. These results reinforce the conclusions of recently reported experiments conducted on the OMEGA EP laser facility at the Laboratory for Laser Energetics at the University of Rochester, NY [36]. They demonstrate that it is possible to use high-power short pulse lasers to carve a long-lasting, low-density channel through large-scale length, fusion-relevant plasmas in the direction of the density gradient via relativistically enhanced channelling: the Habara–Kodama–Tanaka (HKT) ‘super-penetration’ mechanism [37,52]. This now underpins future experiments on LMJ-PETAL or OMEGA laser facility. In the OMEGA EP campaign, Ceurvorst *et al.* [37] successfully optimized channel formation and established the feasibility of stable channels at lower intensities (*I* ≈ 10^18^ W cm^−2^) which corresponds well to the parameters of the NIF ARC system.

**Figure 2.**
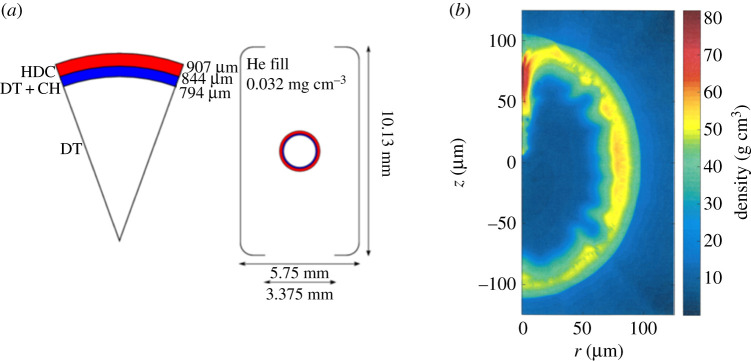
(*a*) The schematic of the wetted foam target design as used for NIF shot 160421 and (*b*) the density profile of this target at peak compression, as calculated from xRAGE simulations. (Online version in colour.)

Having thus established the feasibility of both NIF ARC and LMJ-PETAL forming evacuated channels within fusion plasmas via the relativistic self-focusing HKT mechanism [37,52], we now turn to a more specific consideration of how one expects auxiliary heating to boost the yield of inertial fusion implosions. For the purposes of stable channel formation in fusion plasmas, low convergence ratio implosions are desirable due to their robustness against hydrodynamic instabilities and low-mode asymmetries. We choose to focus on a recent development in low-convergence ratio targets: ‘wetted foam’ targets [46,47]. The capsule design and fill tube, as used for shot 160421 at the National Ignition Facility can be seen in figure 2*a*. This was a sub-ignition scale target and was one of a number designed to study the performance of implosions with different convergence ratios. The primary difference between this design and that of typical central hot spot targets is the use of a thin plastic foam layer that has been wetted with a DT liquid [48,49]. These designs have been shown to display remarkable agreement between experimental data and one-dimensional simulations [50,53]. A typical simulated density profile for these implosions is shown in figure 2*b*. The hot-spot core diameter for ‘wetted foam’ implosions is consistently a factor of two larger than implosions of targets comprising the HDC ablator layer and a cryogenic DT fuel layer [50]. These hot spots are larger because of the significantly higher initial vapour pressure in the capsule centre that, in turn, provides more mass for the hot spot at peak compression.

**Figure 3.**
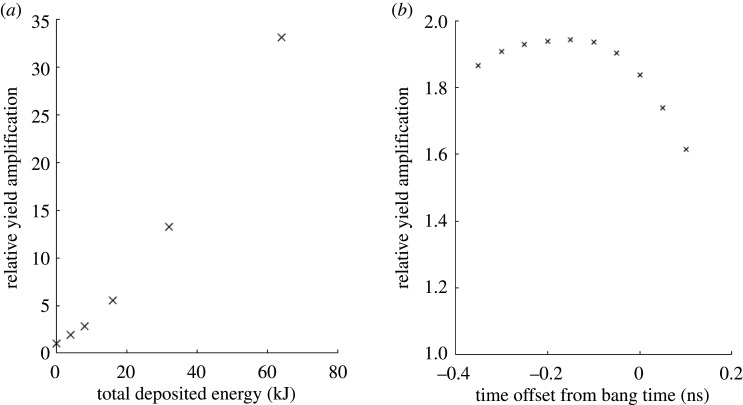
xRAGE simulations with NIF shot 160421 parameters showing the relative amplification of the fusion yield due to auxiliary heating versus (*a*) the total energy deposited into the background electron for a deposition 150 ps before the bang time and (*b*) the offset between the deposition time and the bang time for a total deposition of 4 kJ.

Low convergence ratio ‘wetted foam’ implosions naturally lend themselves to scalable, predictable fusion outputs which are modelled here with the xRAGE code [50]. Our simulations follow the post-shot modelling procedure outlined by Haines *et al.* [50] and include accurately detailed, and well-resolved models for the capsule fill support tent, surface defects and drive asymmetry [53,54]. It has been shown that by including these models, xRAGE is able to accurately reproduce experimental observables [47]. With this success to lend confidence in our simulations, the energy deposition of the electron beams was modelled as uniform across the DT gas over a period of approximately 0.5 ps. The amount of energy deposited, and the offset from the deposition time and the bang time (or time of peak compression) were varied across a number of simulations, the results of which are shown in figure 3. It is clear that the relative amplification of fusion yield is reasonably insensitive across the 300 ps before the bang time. It is anticipated that this scheme should also be relatively insensitive to variations in laser pointing. The reason for this is twofold. Firstly, it is possible to aim laser-formed channels precisely and mitigate both the hosing and filamentation instabilities [37]. Secondly, although the growth rate for the instability that seeds energy into the Langmuir wave is maximal for the case of orthogonal electron beams, Ratan *et al.* [45] demonstrated that Langmuir waves are seeded over the full range of intersection angles including the least optimal case of counter-propagating beams. This implies that the auxiliary heating mechanism should be robust against timing jitters between laser beams that makes this scheme experimentally viable for testing on facilities such as NIF and LMJ. Further, it is apparent that as an ever-increasing number of facilities employ petawatt and multi-petawatt laser technologies, auxiliary heating offers significant opportunity to study the increase of the fusion yield with these new laser beam parameters. Finally, we note that similar deposited energy requirements to those of fast ignition are required. Consequently, the development of plasma optics and plasma amplifiers is an urgent requirement for this scheme as well as a key component of the European Roadmap.

## 4. Benefits of investing in inertial fusion science and technologies

Realizing inertial fusion energy will be transformative for humanity because it allows us to envisage strategies for minimizing the human causes of climate change, while still lifting the remainder of the world’s population out of absolute poverty. There are no long-lived radioactive by-products if the reactor chamber materials are carefully selected. An inertial fusion energy reactor is compatible with the existing electricity infrastructure distribution and generation grid. The process is inherently safe from nuclear meltdown, each nation can have the security of an abundant long-lasting fuel supply, and helium-exhaust emission is friendly to the environment.

Industrial spin-offs often accompany technological advances and fusion break-throughs are no different. For example, investment in the laser-related photonics industry will generate many new spin-offs and highly skilled jobs. The Institute of Physics estimates that the photonics industry in the UK contributes £13 billion annually to the economy. The Institute has recently pointed out that the industry’s status as an enabling technology for multiple uses across numerous sectors means that it still lacks a singular champion the importance of photonics. In addition, the rapid technological advances within the photonic industry provides a powerful insight into the forthcoming photonics revolution.

Just one example of the potential of industrial spin-offs from investments in fusion and accelerator science is that of our new £6.75M spin-out company Living Optics, launched in March 2020, from Oxford Physics. Here, we are utilizing the tremendous power of hyperspectral imaging, together with chirped laser pulses, to obtain movies comprising a series of femtosecond-resolution images of ultra-fast phenomena on a single shot. Our plan is to deploy this new technology across a number of industrial sectors, from defence, fundamental phenomena in physics, chemistry and medical imaging through to the space sector.

In addition, inertial fusion-related research promises further significant improvements in thermal neutron source brightness, even beyond those expected from the European Spallation Source (ESS) when it is fully commissioned in 2025 [55] Thermal neutron scattering has found applications across the natural sciences, from condensed matter physics through to biochemistry and the life sciences. Increased source brightness provides greater signal to noise and therefore more precise diffraction patterns to determine the structure of the new materials under study.

The technical challenges of generating high repetition rate laser drivers at the required energy to target should not be underestimated, nor should the delivery of safe day-to-day operations of these machines for forefront science and technology purposes. We envisage that a staged approach that provides orders-of-magnitude increases in drive energy to target, building upon already existing diode-pumped solid-state lasers (e.g. 2 kJ–20 kJ–200 kJ–2 MJ and 20 MJ) [56], will allow industrial partnerships to develop naturally and, at the same time, provide both national laboratories and industry the time to acquire the vital skills in managing and operating these machines. A UK-XFEL that combines ultra-bright X-ray sources with such a compression facility is a very attractive combination. Also, a new broad-band compression laser facility, one that is able to minimize laser-plasma instability growth [57,58], is an attractive opportunity. In parallel, pulsed power wire array Z-pinches, magnetic liners and ArF excimer lasers need to continue their development path.

The manufacture of high-quality cryogenic targets for multi-Hz operations is also a formidable challenge. These require the development of new manufacturing methods that combine tritium breeding and extraction from the blanket that surrounds the target, precision assembly and quality control, as well as optimum delivery to the chamber centre. The development of precision robotics, remote diagnostics and control systems for the fusion reactor, along with the development of fusion reactor materials, are common themes with our magnetic confinement fusion colleagues. Such considerations reinforce the conclusions that this is a multi-decadal endeavour.

## 5. Summary

In summary, we have discussed the preparations for a European Research and Development Roadmap for a Demo Reactor. Disparate length- and timescales involved in many aspects of inertial fusion research makes them both challenging for physicists and fascinating in equal measure. The obstacles are difficult to surmount, from both a technological and a scientific viewpoint. Nonetheless, there are promising new solutions to these challenges that are under active investigation by our consortium. We have discussed progress in the conventional central hot-spot scheme and in the fast ignition inertial fusion approach. In addition, we have presented new calculations in the auxiliary heating scheme for augmenting existing fusion programmes whereby additional energy is provided to the hot spot by crossing relativistic electron beams at the position and time of peak compression. By considering low convergence ratio implosions, we have taken advantage of their robustness against hydrodynamic instabilities and low-mode drive asymmetries to establish these implosions as ideal scenarios for stable channel formation in the coronal plasma, as reported elsewhere in this special issue by Spiers *et al.* [38]. We have demonstrated that the increase in fusion yield from this auxiliary heating approach in a sub-ignition implosion design has been well characterized on the National Ignition Facility and is therefore available for experimental verification. Further, we posit that this augments the central hot-spot scheme in a complementary manner to fast ignition, thereby providing an additional and attractive route towards realizing the full potential of inertial fusion. Experimental and theoretical progress on this scheme, concentrating on future developments of plasma optics and plasma amplifiers with advances in laser, target and materials technologies, along with the other alternative approaches such as direct drive, shock ignition and magnetic-assisted compression, will inform the European Roadmap.

## Data accessibility

This article has no additional data.
